# A Compact LIF Spectrometer for in-Field Operation in Polar Environments

**DOI:** 10.3390/s21051729

**Published:** 2021-03-03

**Authors:** Lorenzo Palombi, Valentina Raimondi

**Affiliations:** “Nello Carrara” Institute of Applied Physics—National Research Council, I-50019 Sesto Fiorentino, FI, Italy; l.palombi@ifac.cnr.it

**Keywords:** spectrometer, laser-induced fluorescence, polar environment, biological soil crusts, microorganisms

## Abstract

We present a compact laser-induced fluorescence (LIF) spectrometer prototype (SFIDA–405) designed for in-field operation in polar environments. It uses 405 nm excitation to acquire LIF spectra in the 450–930 nm spectral range on a solid surface via an optical-fiber coupled measurement head. The prototype (battery powered; module + measurement head weight: <1.6 kg) is controlled via a military-grade smartphone and has a limit of detection for chlorophyll better than 5 ng/cm^2^. The instrument was successfully tested during two summer field campaigns in the Arctic (Svalbard Islands) and Antarctic (Southern Victoria Land) regions for studying biological soil crusts. To the best of the authors’ knowledge, this represents the first LIF spectrometer used in situ in Antarctica to acquire LIF spectra directly on biological soil crusts. Finally, the paper also suggests the use of the SFIDA–405 prototype for different application fields.

## 1. Introduction

The availability of field-portable instrumentation is a key factor in environmental studies. This becomes even more crucial for the study of biological organisms, since the laboratory is usually a harsh environment for their conservation, as outdoor-like conditions are difficult to replicate. Typically, measurements carried out in the laboratory cannot be fully representative of the actual conditions in the field.

Field-portable fluorometers such as Pulse-Amplitude Modulation (PAM) fluorometers have been routinely used in basic and applied photosynthesis research ever since the introduction of the first chlorophyll (Chl) fluorometer [1]. They are mainly used to measure the relative Chl fluorescence quantum yield, although recent models can also provide data on other photosynthesis parameters [2]. In the mid 1990s, other portable fluorometers were developed for diverse applications, from agro-forestry to environmental microbiological studies. Most of them used a Light Emitting Diode (LED) as an excitation source or performed the fluorescence acquisition in a limited number of channels [3,4]. Recently, the miniaturization of electro-optical components and the increasing availability of commercial miniaturized spectrometers have paved the way for the in-house development of very compact, low-cost fluorescence spectrometers that are able to detect fluorescence spectra in the full visible spectral range and are tailored for different in-field applications [5,6,7,8,9]. A portable fiber-probe fluorescence detection system using an UltraViolet (UV) LED emitting at 365 nm as an excitation source was developed for the inspection of the ripening stages in horticultural and agricultural products. Although tested only in the laboratory, the system was designed in order to be suitable for in-field operation [6]. A field-portable Laser-Induced Fluorescence (LIF) system was constructed for the fast acquisition of in situ Colored Dissolved Organic Matter (CDOM) fluorescence data without sample filtering [7,8]. A similar instrumental concept was adopted to construct a portable hyperspectral laser fluorometer for coastal and inland water monitoring [9], while a two-channel system with laser excitation at 405 nm was specifically developed for detecting cyanobacteria blooms [10]. A portable, tripod-based system for measuring the fluorescence signal of Chl and phycoerythrobilins by means of a double excitation wavelength was developed and tested on samples of snow and supraglacial ice from a glacier in the Svalbard Islands [11,12]. A similar principle was also used for the development of an LIF spectrometer with a 266 nm excitation to perform fast virological analyses [13]. A very compact broadband fluorometer was recently constructed and its use was demonstrated for various applications, from capillary electrophoresis separation to flow cytometry [14].

This paper presents the first portable, handy LIF spectrometer—called the SFIDA–405—specifically designed, constructed, and extensively used to acquire in situ measurements of LIF spectra on solid surfaces in polar environments. Besides the technical characteristics of the prototype, this paper reports the typical LIF spectra acquired in situ on different environmental targets (biological soil crusts, algal mats, rocks) during two polar (Arctic and Antarctic) measurement campaigns. To the best of the authors’ knowledge, this represents the first LIF spectrometer used in situ in Antarctica. Finally, the paper suggests the use of the SFIDA–405 prototype in different application fields.

## 2. Instrumentation Description

**Figure 1:**
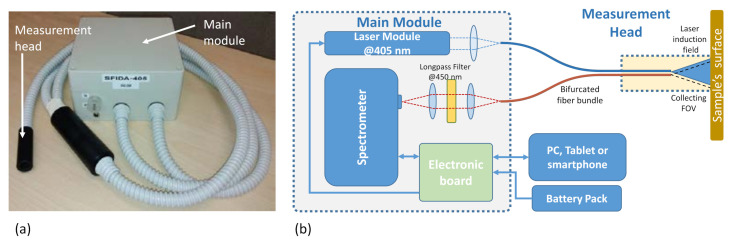
SFIDA–405 prototype: (**a**) main module and measurement head and (**b**) block diagram.

The SFIDA–405 prototype (Figure 1) is an optical fiber-coupled compact LIF spectrometer; it was specifically designed to be transportable in a small backpack and suitable for day-long, outdoor use in polar environments. The main module (Figure 1a) is compact, battery-powered, and optically coupled to a small, handy measurement head. The latter is placed in contact with the surface of interest to acquire the LIF spectra. The instrument can be controlled via either a laptop or tablet or smartphone using in-house developed software.

A block diagram of the prototype is shown in Figure 1b. The laser source is a Continuous Wave (CW) laser diode emitting at 405 nm. The wavelength was chosen as a compromise between the following factors: (1) miniaturized laser models available on the market and (2) the excitation of a wider range of fluorophores, yet with an efficient excitation of Chl. The output power at the measurement head is about 50 mW. The beam is focused on the tip of one optical fiber (induction fiber) of a bifurcated bundle (fused silica, 600 µm core diameter) and propagated up to the other tip of the fiber located inside of the measurement head. The backscattered laser radiation and the fluorescence signal emitted by the surface are collected by the second optical fiber (collecting fiber) of the bifurcated bundle. The measuring head is mechanically constructed so as to maintain a fixed distance between the optical fiber tips and the surface to be investigated. The default operating distance between the optical fiber tips and the surface was set to 11 mm, corresponding to a measured surface of the 5 mm diameter; this was regarded as the best compromise between the signal intensity and the measurement of a statistically significant surface area. However, this operating distance can be varied easily, if required, depending on the characteristics of the target to be investigated. The head is arranged so that the area illuminated by the laser can be considered coincident with the area seen by the collecting fiber. At the other end of the collecting fiber, an optical system constituted by a collimating lens, a dichroic optical filter, and a focusing lens is used to spectrally filter the light and focus it on the entrance slit of a compact spectrometer (OceanOptics, Orlando, FL, USA; USB2000+ model). A dichroic longpass optical filter with a cut-on wavelength at 450 nm (Thorlabs, Newton, NJ, USA; FEL0450 model) is used to reject stray light and to prevent saturation issues caused by the elastically backscattered laser light. A custom electronic board ensures the full control of the spectrometer operational parameters and laser synchronization. The battery pack is a standard powerpack commonly used to charge electronic devices (PNY Technologies Europe, Mérignac cedex, France; T10400 model, 5 V, 10400 mAh, max 2 A) and ensures over 30 h of instrument operation. All the parameter settings and data acquisitions are fully controlled via an in-house developed software. The instrument can be fully controlled by means of a military-grade smartphone.

The components were chosen according to the following main criteria: (1) pros and cons analysis of the technical specifications of a range of compact spectrometers and miniaturized lasers available on the market, and (2) light weight and ruggedness. It is to be noted that the detection limit of Chl was not the only objective pursued in the development of the prototype, but also, for example, the capability of detecting a wider range of fluorophores, yet with an efficient excitation of Chl.

During in-field operation, the prototype is housed inside a small backpack padded with insulating material. The backpack was chosen among those commercially available for carrying camera equipment. The temperature of the main module is continuously monitored and an alarm is sent to the user via software if it falls below a certain preset threshold. Since the smartphone ensures full control of the prototype, there is no need to extract the main module from the backpack during operation. The smartphone, which together with the measurement head is the only part exposed to external environmental conditions, guarantees full operation down to temperatures of −25 °C.

The LIF spectrum is acquired in the spectral range between 450 and 930 nm with a mean spectral sampling of 0.33 nm and a mean spectral resolution, Full Width Half Maximum (FWHM), of 2.0 nm. The LIF spectrum is obtained as the difference between two spectra acquired using the same instrumental parameters: the first spectrum is acquired while the laser is on and the second one is acquired with the laser off and records the background spectrum. The subtraction of the background spectrum removes the spurious contributions introduced by the spectrometer baseline and by any residual ambient light reflected by the surface. The latter, however, is minimized thanks to the mechanical arrangement of the measuring head that protects the examined surface from being illuminated by the ambient light. Typically, the acquisition of an LIF spectrum requires from one to several tens of seconds, depending on the intensity of the signal and the signal to noise ratio (SNR) required by the specific application.

**Figure 2:**
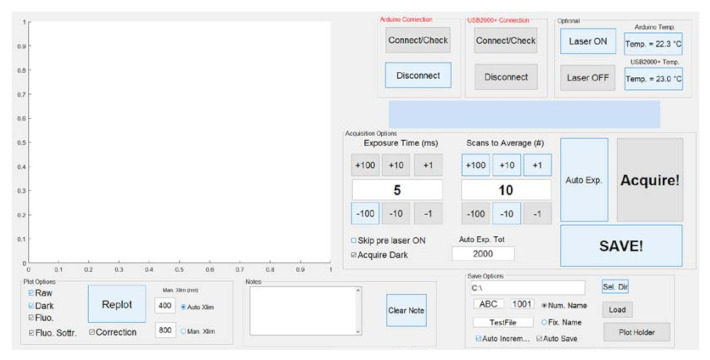
SFIDA–405 graphical user interface.

A handy graphical user interface (Figure 2) is used to set all the required parameters, control data acquisition, and display the spectrum as soon as it is acquired. In order to increase the quality of the acquired data, the software permits setting both the exposure time and the number of consecutive exposures that are averaged to obtain the final spectrum. The setting of the exposure time permits exploiting the acquisition dynamic range of the spectrometer at its best, depending on the fluorescence efficiency of the examined surface.

An additional functionality available in the SFIDA–405 software is the autoexposure function. This was implemented in order to automatically optimize the quality of the LIF spectra, given a total exposure time set by the user. This function automatically evaluates the optimal exposure time according to the signal acquired using a very short exposure. The number of consecutive exposures to be averaged to obtain the final spectrum is then calculated in such a way as to match the total exposure time set by the user. The autoexposure function can be particularly useful during in-field operation in order to both limit, as much as possible, the arbitrary selection of parameter settings by the user and minimize the time needed to acquire measurements on heterogeneous surfaces characterized by very different fluorescence efficiencies.

The SFIDA–405 main technical data are summarized in Table 1.

## 3. Instrument Performance

The performance of the SFIDA–405 prototype was evaluated in the laboratory by acquiring the LIF spectra on different dilutions of a stock solution containing Chl a and Chl b (Chl a: 2.33 mg/mL; Chl b: 0.96 mg/L; total Chl: 3.29 mg/L). The stock Chl extract was obtained from spinach leaves using 80% acetone (80% acetone, 20% water (*v*/*v*)) as s solvent. The Chl a and Chl b concentrations in the stock extract were evaluated by spectrophotometric absorption measurements [15]. A total of 31 different dilutions, ranging from a minimum total Chl concentration of 0.5 µg/L to a maximum of 823.3 µg/L, were measured by filling a UV-fused quartz glass cuvette with a 10 mm optical path length. An additional measurement on pure water was acquired and used as a blank. Measurements were obtained by placing the SFIDA–405 measurement head on the side of the cuvette. The total integration time was set to 10 s per measurement.

**Figure 3:**
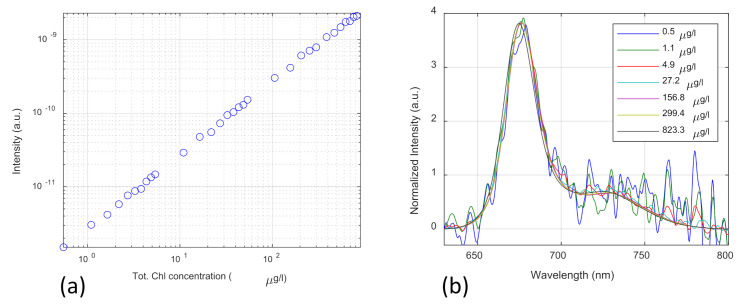
Laser-Induced Fluorescence (LIF) spectra for different Chl concentrations: (**a**) the log-log scale scatterplot of the fluorescence intensity (mean fluorescence signal in the 630–800 nm spectral range) as a function of the total Chl concentration; (**b**) the subset of the measured LIF spectra for different Chl concentrations. The intensity was normalized to the standard deviation.

The linearity of the instrumental response was evaluated by applying a linear regression to the mean fluorescence signal in the 630–800 nm spectral range as the dependent variable and the total Chl concentration as the explanatory variable. A very good linearity, with an adjusted coefficient of determination of R2 = 0.9981, was obtained. The log-log scale scatterplot of the signal as a function of the total Chl concentration is shown in Figure 3a.

The limit of detection (LOD) of the instrument was evaluated by comparing the spectral distribution of the fluorescence spectra at the different total Chl concentrations. Figure 3b shows a subset of the measured fluorescence spectra. The intensities were normalized to the standard deviation in order to facilitate the comparison of the spectral distributions. The total Chl LOD, estimated as the lowest concentration that can be confidently distinguished from the blank, is 0.5 µg/L. Given the 10 mm optical path, this value corresponds, on a surface, to a total Chl density of 0.5 ng/cm^2^.

In addition, the system operation was tested in the laboratory at a temperature range of 4 to 40 °C. The only parameter that was found to be temperature-dependent was the baseline signal of the spectrometer. However, since the acquisition procedure of the fluorescence spectrum already included the subtraction of the background spectrum, the former already provided an automatic correction of baseline variations. The emission wavelength of the laser diode was also tested in the laboratory at a temperature range of 4 to 40 °C. The observed wavelength variations were less than 1.5 nm. Since the absorption spectra of the fluorophores of interest in this wavelength range can be considered constant, these variations were regarded as not meaningful.

## 4. In-Field LIF Measurements in Polar Environments

The SFIDA–405 prototype was first tested during an eight-day measurement campaign in Ny-Ålesund (Svalbard Islands) in August 2014. During this first field campaign, the instrument was used in more than 50 different spot sites reached by boat and on foot in the Kongsfijorden area during the campaign. Subsequently, the instrument was extensively used during a measurement campaign in Northern Victoria Land, Antarctica, which was carried out under the Italian National Research Program in Antarctica. The campaign lasted from 20 November to 31 December 2015, and it also included a five-day remote camp in the internal Dry Valleys without any laboratory facilities. During the Antarctic measurement campaign, the scientific personnel and instrumentation were transported aboard a helicopter in selected sites, situated mainly in the coastal areas of Northern Victoria Land. In the whole, the SFIDA–405 prototype was used in more than 25 different measurement sites and about 1000 LIF measurements were collected on-site. Air temperatures during the in-field operation typically ranged from −5 to +2 °C.

### 4.1. Biological Soil Crusts

During both measurement campaigns, the SFIDA–405 prototype was mainly used to detect biological soil crusts on-site and to characterize their pigment content, with an emphasis on those crusts at a very early stage of growth. In general, the soil crusts examined at the selected sites in the Ny-Ålesund area during the first test campaign presented a higher variability of pigment content and, in several cases, could reach very advanced stages of development. The soil crusts investigated at the Antarctic sites in Northern Victoria Land were often in an early stage of development, although at some sites well-developed crusts could also be found.

**Figure 4:**
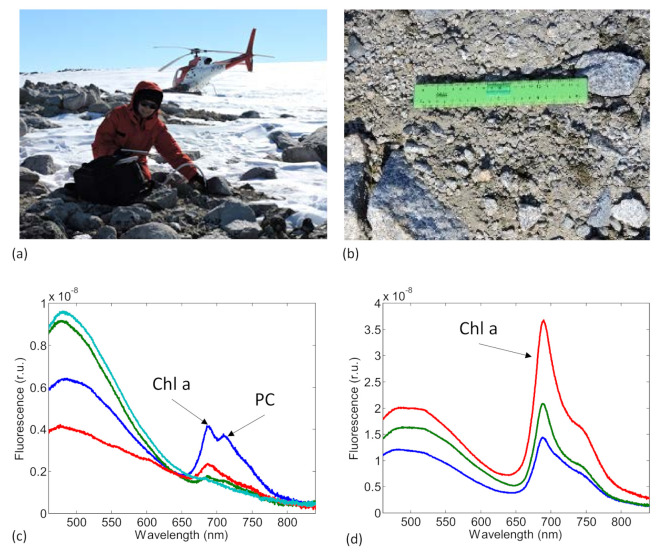
In situ LIF spectra on biological soil crusts acquired during the Antarctic campaign. (**a**) SFIDA–405 prototype during in field operation at Prior Island, Antarctica. (**b**) Terrestrial microbiological crust at an early stage of development. (**c**) LIF spectra detected in the field on different points of the crust shown in (**b**). (**d**) LIF spectra on different points of a terrestrial microbiological crust in an advanced stage of development.

Figure 4 shows the experimental conditions and typical LIF spectra obtained from microbiological crusts at a measurement site at Prior Island, Northern Victoria Land, Antarctica. The picture in Figure 4a shows the experimental conditions in the field during the acquisition of the LIF spectra with the SFIDA–405 prototype; the LIF spectra were acquired by placing the measurement head in contact with the crust. The measurement head was connected to the sensor only via an optical fiber (Figure 1). In this way, there was no need to expose the sensor to the external conditions. The main module was kept inside the insulated small backpack all through the measurement time, while instrumental control was granted by means of a rugged smartphone. Figure 4b,c show, respectively, a crust at an early stage of development and the corresponding LIF spectra acquired in different spots; here, the fluorescence spectral distribution is typical of a phycocyanin (PC)-containing biological crust because of an additional fluorescence peak at 710 nm. Figure 4d shows the spectra acquired on a crust in an advanced stage of growth; in this case, the spectral distribution is clearly different and dominated by the presence of Chl with a fluorescence peak at 690 nm.

### 4.2. Algal and Cyanobacteria Mats

The SFIDA–405 spectrometer was also used to detect and identify the pigment content of algal mats close to a dry drainage stream in the area at Lake Bonney, located in the Dry Valleys of Antarctica.

**Figure 5:**
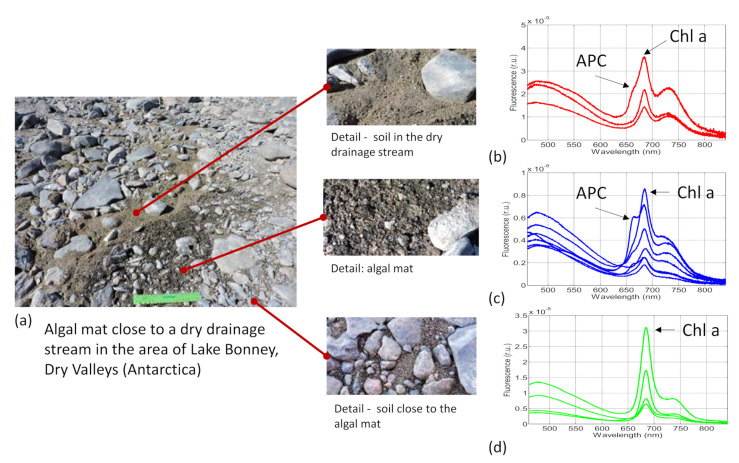
LIF spectra on algal mats close to a dry drainage stream (Lake Bonney area at Dry Valleys, Antarctica). (**a**) Picture of the examined area with algal mats. (**b**) Detail of the soil in the drainage stream and relevant LIF spectra. (**c**) Detail of the algal mat and relevant LIF spectra. (**d**) Detail of the soil close to the algal mat and relevant LIF spectra.

Figure 5a shows the area featuring a dry drainage stream with an algal mat. LIF spectra were acquired in several spots. Figure 5b shows the LIF spectra acquired on soil in the drainage stream; the spectra show an intense Chl fluorescence, but also a typical cyanobacterial component (additional fluorescence contribution at 660 nm due to allophycocyanin (APC)). The same spectral features were found in the algal mat (Figure 5c). In this case, the allophycocyanin contribution at 660 nm is definitely apparent. Thanks to the high sensitivity of the SFIDA–405, an intense Chl fluorescence signal was also detected in several spots on the soil in which there was not any evidence of biological growth by visual examination (Figure 5d).

### 4.3. Natural Rocks

**Figure 6:**
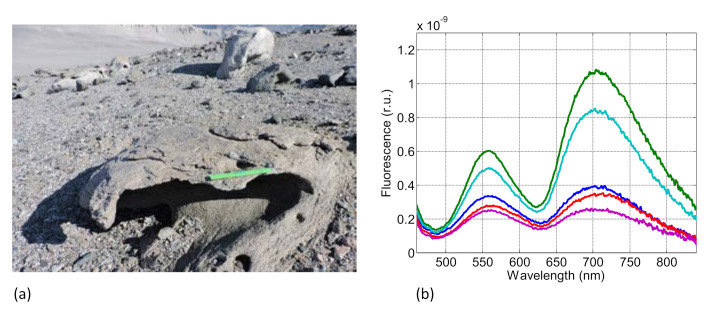
LIF spectra on a weathered magmatic rock (Lake Joyce area, Dry Valleys, Antarctica). (**a**) Magmatic rock on which the LIF spectra were acquired. (**b**) LIF spectra acquired in different spots of the rock surface.

Besides biological soil crusts, the SFIDA–405 instrument was also used to detect the fluorescence properties of natural rocks directly in situ. As an example, Figure 6 reports the LIF spectra acquired on a weathered rock in the Lake Joyce area in the Dry Valleys, Antarctica [16]. Figure 6a shows a magmatic rock (monzodiorite) on which the measurements were acquired. Figure 6b shows the relevant LIF spectra. The two fluorescence bands at 560 and 710 nm are compatible with the presence of quartz [17,18].

## 5. Other Application Fields

The versatility of the prototype and its technical characteristics make it suitable for use in several other application fields, from non-invasive fluorescence diagnostics of cultural heritage to industrial processing control and environmental monitoring.

Here, we report an example of an on-site analysis of the biological growth on stone cultural heritage. LIF spectra, actually, are a very sensitive tool to detect the presence of photoautotrophic organisms on a stone surface, well before they are visible to the naked eye. The spectral bands can provide useful information on the pigment content of the biological patina and thus a preliminary classification of the species present on the surface. Such information is valuable for understanding the biodeterioration process and for outlining the most suitable conservation strategies.

Biological growth on the external walls of the *Santa Maria della Bruna* cathedral in Matera, Italy, was investigated by using the SFIDA–405 prototype. The external walls of the cathedral showed an apparent dark alteration of the limestone in their lowest part, up to 6–8 m above the ground. The origin of this dark alteration of the limestone, whose presence was already known at the beginning of the XX century, was the object of a multidisciplinary study [19].

**Figure 7:**
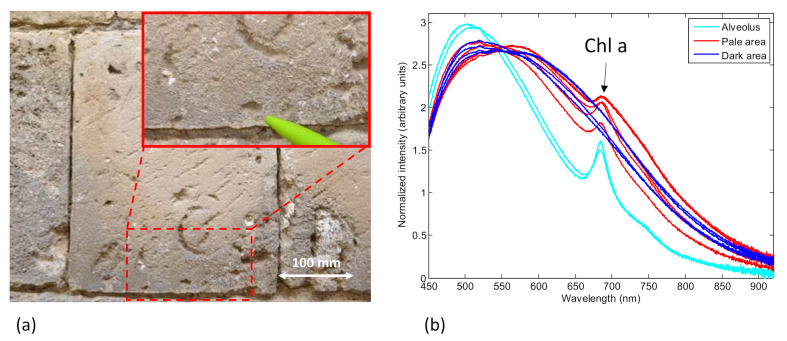
On-site LIF analysis of the biological growth on a stone cultural heritage. (**a**) Ashlar of the external walls with a dark alteration; (**b**) LIF spectra in different spots of the same ashlar. The spectra are normalized to their standard deviation.

One of the ashlars with areas affected by this dark alteration is shown in Figure 7a. LIF measurements were acquired on several spots of both the upper (clear) and lower (dark) part of this ashlar. The LIF spectra are shown in Figure 7b: the spectra are intensity-normalized to their standard deviation to underline their different spectral distribution. The spectra of both the clear (red line) and dark areas (blue line) have a broad fluorescence band due to the substrate (limestone) with two main contributions of fluorescence at about 510 and 610 nm. However, only the spectra acquired on the clear area (red line) show an additional fluorescence contribution at 685 nm, with a spectral shoulder at about 740 nm, which is typical of biological patinas containing Chl *a* on stone monuments [20,21]. Dark area spectra did not show any presence of Chl *a*. Similar results were found examining other ashlars with the same dark alteration.

The results suggest the presence of an extended biological patina containing photoautotrophic microorganisms exclusively in the clear part of the ashlar. Interestingly, the only spot of the ashlar’s dark area where the spectra showed the typical peak of Chl *a* fluorescence was in correspondence to an alveolus of the limestone (inset in Figure 7a). The spectra detected in the alveolus, with their distinctive fluorescence peak at 685 nm, are shown in Figure 7b (cyano lines). The LIF analysis led us to conclude that the black alteration had an antagonistic role with respect to the photoautotrophic microorganisms.

## 6. Conclusions

In this paper, we presented a rugged, handy prototype of a LIF spectrometer designed for acquiring fluorescence spectra in harsh environments. The instrument was successfully used over the course of summer measurement campaigns in the Svalbard Islands and in Northern Victoria lLnd, Antarctica. The instrument features a remarkable ability in the detection of low Chl concentrations (down to 5 ng/cm^2^ of total Chl) and provides a linear response over a wide range of concentrations (three orders of magnitude). The SFIDA–405 instrument, which was battery powered and smartphone-connected for easy control in the field, was used for the detection and spectral characterization of biological soil crusts in different sites, showing a very high sensitivity for the detection of biological growth on the soil at a very early stage (pre-visual) of development. To the best of the authors’ knowledge, this represents the first LIF measurements acquired in situ in Antarctica. Thanks to its versatility and technical characteristics, the SFIDA–405 prototype is also suitable for non-invasive LIF diagnostics in other application domains that require on-site operation, such as immovable cultural heritage diagnostics and environmental studies in agroforestry and terrestrial applications. As a future development, we are exploring the feasibility of coupling the system to a scanning system in order to acquire a hyperspectral fluorescence image of the sample surface.

## Figures and Tables

**Table 1:** SFIDA–405 main technical data.

Parameter	Values
Laser excitation wavelength	405 nm
Excitation power	50 mW
Induction and collection Field of View (FOV)	25.4° (full angle); Ø 5 mm @ 11 mm
Fiber bundle length	2 m
Longpass cut-on wavelength	450 nm
Spectral range	From 450 nm to 930 nm
Mean spectral sampling	0.33 nm
Mean spectral resolution (Full Width Half Maximum—FWHM)	2.0 nm
Integration time setting (single spectrum)	From 1 ms to 65 s
Additional SW functionalities	Automatic background subtraction Realtime spectra display Autoexposure Temperature monitoring
Weight (main module + measuring head)	1.6 kg
Dimensions (main module)	160 mm × 160 mm × 90 mm
Power consumption	<1 W

## Data Availability

The data presented in this study are available on request from the corresponding author.
